# Left atrial epicardial adipose tissue is closely associated with left atrial appendage flow velocity in patients with nonvalvular atrial fibrillation

**DOI:** 10.1038/s41598-022-13988-3

**Published:** 2022-06-24

**Authors:** Yameng Shao, Lei Chen, Changjiang Xu, Beibei Gao, Dongdong Zhang, Chuanyi Sang, Chaoqun Zhang

**Affiliations:** 1 Department of Cardiology, The Affiliated Hospital of Xuzhou Medical University, 99#, Huaihai West Road, Xuzhou, 221002 China; 2 Department of Cardiology, The Affiliated Huaian No. 1 People’s Hospital of Nanjing Medical University, 6#, Beijing West Road, Huaian, China

**Keywords:** Cardiology, Medical research, Risk factors

## Abstract

Epicardial adipose tissue (EAT) can play an important role in the occurrence and development of atrial fibrillation and stroke. In this study, we explored the relationship between left atrial epicardial adipose tissue (LA-EAT) and left atrial appendage flow velocity (LAA-FV) in patients with nonvalvular atrial fibrillation (NV-AF). A total of 145 patients with NV-AF who underwent their first radiofrequency ablation were enrolled. They underwent left aortopulmonary vein computed tomography angiography (CTA) and transesophageal echocardiography (TEE) before AF ablation. Left atrial (LA) electroanatomical mapping was performed intraoperatively to assess left atrial voltage. Univariate regression analysis showed that LAA-FV was lower in patients with a low voltage zone (LAA-FV; 35.02 ± 10.78 cm/s vs. 50.60 ± 12.17 cm/s, P < 0.001). A multiple linear regression model showed that the left atrial low voltage zone (β = − 0.311 P < 0.001), LA-EAT volume (β = − 0.256 P < 0.001), left atrial appendage shape (β = − 0.216 P = 0.041), LAVI (β = − 0.153 P = 0.041), and type of atrial fibrillation (paroxysmal vs. persistence) (β = − 0.146 P < 0.048) were independent predictors of LAA-FV. In NV-AF patients, the increase in LA-EAT volume is related to the decrease in LAA-FV.

## Introduction

Atrial fibrillation (AF) is the most common arrhythmia^1^. Although the management of atrial fibrillation has been continuously improved, there are obvious limitations of antiarrhythmic drug therapy and radiofrequency ablation^2,3^, and the long-term maintenance of sinus rhythm in patients with atrial fibrillation is challenging. Therefore, anticoagulation therapy is very important.

The most important source of embolic stroke in patients with nonvalvular atrial fibrillation (NV-AF) is the left atrial appendage^4^. Many studies have shown that a decrease in left atrial appendage velocity (LAA-FV) is closely related to left atrial appendage mural thrombosis^5,6^. At present, the main measurement method for LAA-FV is transesophageal ultrasound^7^, but this is a semi-invasive approach, and its accuracy is related to the skill level of the operator. During the COVID-19 pandemic, noninvasive tests were recommended to reduce the risk of exposure to the virus^8,9^. Epicardial adipose tissue (EAT) is in direct contact with the surface of the atrium and pulmonary vein and, like the myocardium, is supplied by the coronary artery^10^, which can play an important role in the occurrence and development of atrial fibrillation and stroke through a variety of mechanisms, such as fat infiltration, fibrosis and inflammation^11,12^. Previous studies have shown that myocardial fibrosis is closely related to the velocity of the left atrial appendage^13,14^. However, the relationship between epicardial adipose tissue and left atrial appendage velocity is rarely reported.

With the popularization and development of electrophysiological technology in recent years^15,16^, it is generally accepted that atrial fibrosis can be shown by mapping the low voltage zone through electrophysiological intracavitary voltage mapping^17,18^. In this study, we identified atrial fibrosis by left atrial voltage mapping during radiofrequency ablation to explore the relationship between LA-EAT and left atrial appendage velocity in patients with nonvalvular atrial fibrillation. We hope to find a safe and reliable index to evaluate left atrial appendage function.

## Methods

### Study population

A total of 145 patients with nonvalvular atrial fibrillation who underwent their first radiofrequency ablation at the Affiliated Hospital of Xuzhou Medical University between 2020 and 2021 were selected. All patients underwent CTA and TEE examinations of the left atrium and pulmonary vein before the operation. There were 76 patients with paroxysmal atrial fibrillation and 69 patients with nonparoxysmal atrial fibrillation. All patients were diagnosed with atrial fibrillation by electrocardiogram and/or dynamic electrocardiogram. Paroxysmal atrial fibrillation is defined as atrial fibrillation that lasts less than 7 days and terminates spontaneously, while persistent atrial fibrillation is defined as atrial fibrillation that lasts more than 7 days or requires electrical cardioversion and/or drug cardioversion to terminate. Exclusion criteria: (1) previous catheter ablation of atrial fibrillation or other cardiac surgery; (2) hyperthyroidism; (3) rheumatism and other cardiac valvular diseases; (4) active connective tissue disease; and (5) inability to undergo CTA or CT imaging or inability to obtain complete left atrial appendage data because of contrast medium allergy. This study was reviewed by the Ethics Committee of the Affiliated Hospital of Xuzhou Medical University, and all patients signed informed consent forms.

### Echocardiographic measurements

**Figure 1:**
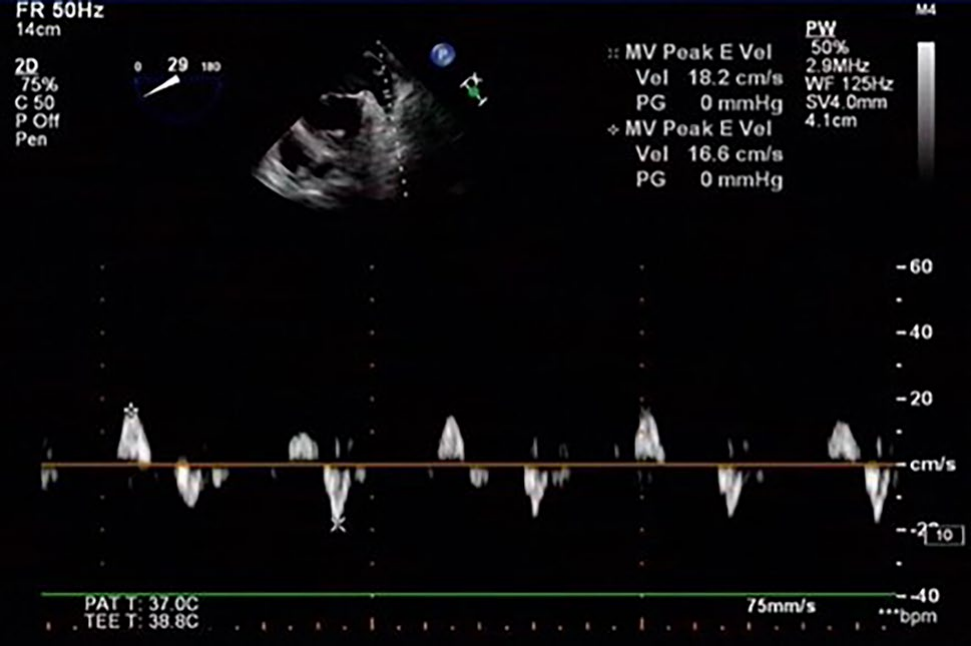
The blood flow pattern of the left atrial appendage in sinus rhythm.

**Figure 2:**
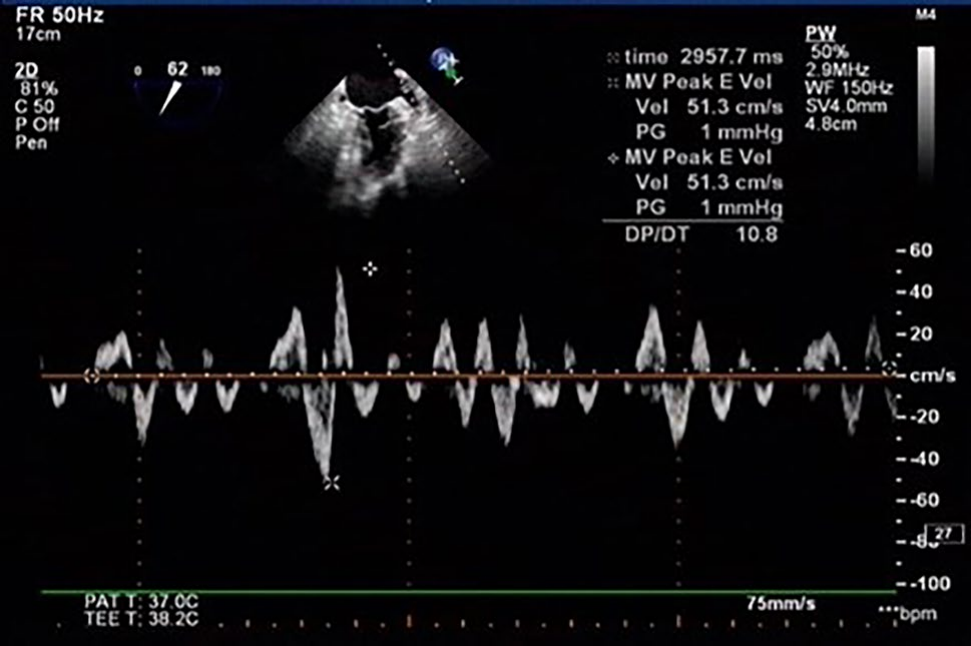
The blood flow pattern of the left atrial appendage in atrial fibrillation rhythm.

Transthoracic echocardiography (TTE) was performed with a Philips EPIQ7c ultrasonic diagnostic instrument, S5-1 probe and probe frequency 1–5 MHz. Parameters such as left ventricular ejection fraction (measured by the biplane Simpson's method) were recorded in detail in the left recumbent position. TEE was examined by PhilipsiE33 color Doppler ultrasonography with an X7-2t transesophageal matrix real-time three-dimensional probe with a frequency of 2–7 MHz. Before fasting for 6–8 h, the ECG was recorded synchronously, the pulsed Doppler sampling volume was placed within 1 pulse near the opening of the left atrial appendage, and the blood flow spectrum of the left atrial appendage was obtained. The left atrial appendage blood flow spectrum was a regular two-way wave in sinus rhythm and an irregular zigzag waveform in atrial fibrillation rhythm. The peak value of the positive wave, that is, the maximum emptying velocity of the left atrial appendage, was recorded in 3 and 10 cardiac cycles, respectively, and the average value was taken as the velocity of the left atrial appendage (LAA-FV) (Fig. 1–2).

### Cardiac computed tomography imaging

**Figure 3:**
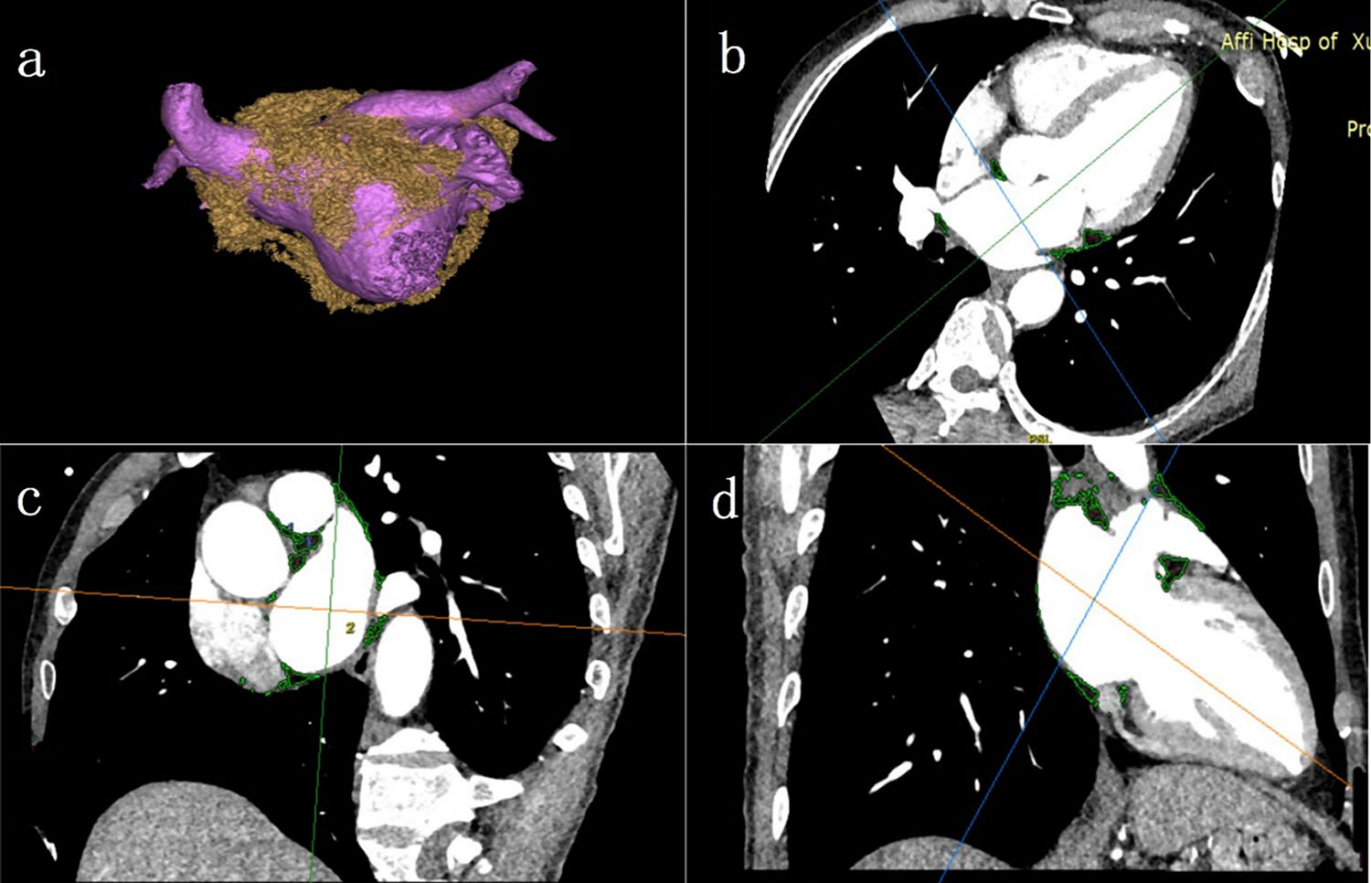
(**a**) The distribution of anterior and posterior left atrial epicardial adipose tissue (LA-EAT) around the left atrium, left atrial appendage and pulmonary veins (yellow represents fat); (**b–d**) the distribution of EAT under multi-planar reconstruction (green represents fat).

Spiral CT (SOMATOM Definition, SIEMENS Germany) was used to obtain CT imaging data. Iohexol (60–80 ml) was injected into the elbow vein at a flow rate of 5 ml/s, and then saline (50 ml) was injected at a speed of 5 ml/s. The contrast agent tracking technique triggered the enhanced scan. Image processing and measurement: All images were reconstructed by retrospective ECG gated reconstruction, slice thickness 0.5 mm, overlapping 0.3 mm, and image postprocessing was performed by an Advantage Workstation 3.2 (GE, USA) workstation. The CT threshold range of the adipose tissue was set to − 50 HU ~ − 200 HU. The left atrial epicardial adipose tissue (LA-EAT) was manually segmented, and the LA-EAT was calculated (Fig. 3). Three-dimensional images of the left atrial appendage and left atrium were obtained by three-dimensional reconstruction of the volume. According to the morphological characteristics of the left atrial appendage, the left atrial appendage was divided into three types: chicken wing type (chicken wing), obvious folding in the proximal or middle part of the main lobe of the left atrial appendage, and nonchicken wing type (nonchicken wing), excluding other forms of chicken wing type. The left atrial volume (LAV) and left atrial volume index (LAVI) were calculated, defined as the left atrial volume divided by the body surface area. All images were measured and evaluated by two independent clinicians using the same measurement methods, and any inconsistencies were resolved through consultation.

### Detection of the low voltage zone (LVZ)

**Figure 4:**
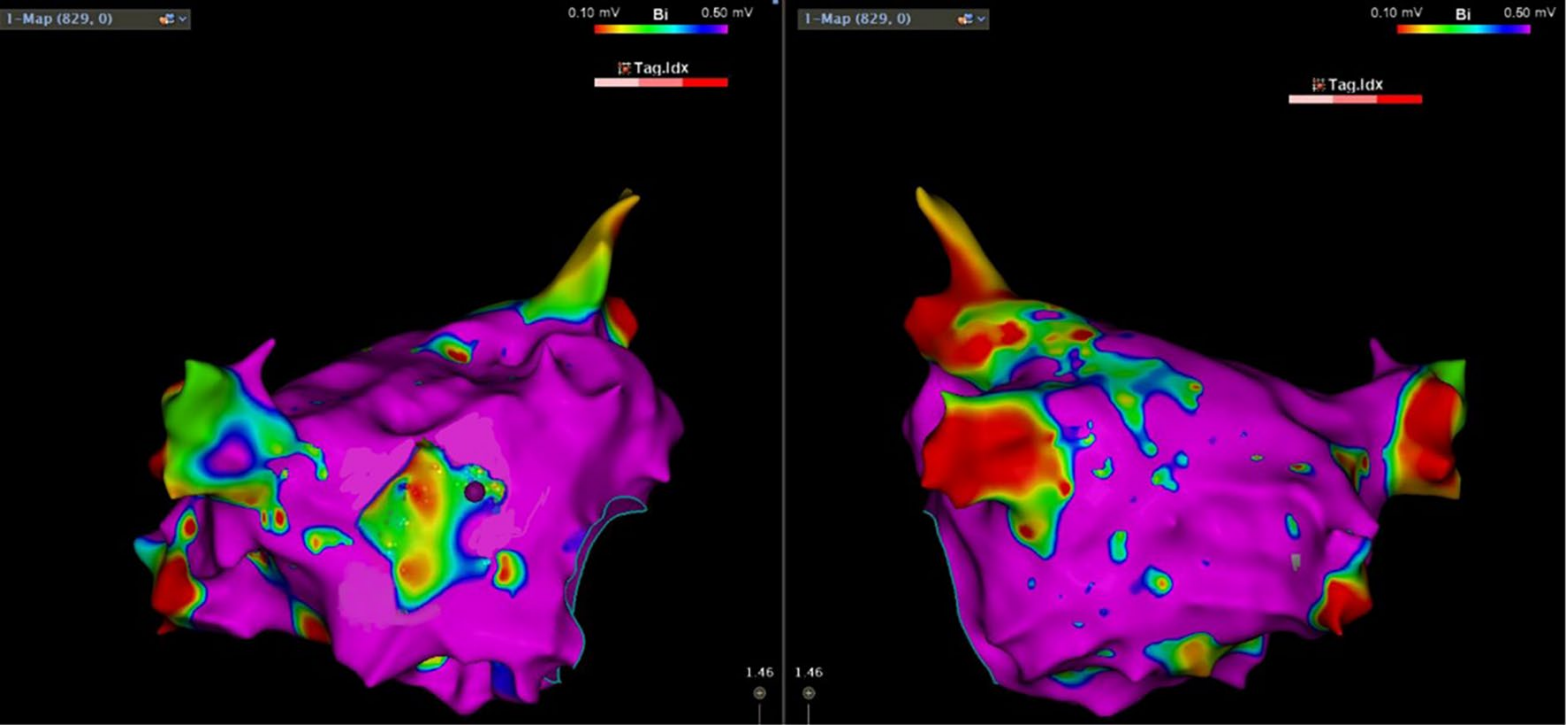
Left atrial (LA) electroanatomical maps created during sinus rhythm, and the location of the anterior wall low voltage zone (LVZ) of the left atrium. Posterior-anterior view (Left); anterior–posterior view (Right).

All patients first underwent circumferential pulmonary vein isolation (PVI). Left atrial voltage mapping was performed directly in patients with paroxysmal atrial fibrillation after PVI. Patients with persistent atrial fibrillation underwent synchronous electrocardioversion if they were still in atrial fibrillation after isolation, and voltage mapping was performed after cardioversion. Voltage mapping was carried out with an ST ablation catheter (Johnson Company, USA), and contact force values of > 5 g for mapping electrograms confirmed adequate endocardial contact. We used ≥ 3 adjacent peak-to-peak bipolar voltage cutoffs of 0.2–0.5 mV to define the low-voltage region to more accurately delineate the LAVI^19^ (Fig. 4).

### Statistical analysis

If the measurement data were normally distributed, they were expressed as the mean ± SD. If they do not conform to a normal distribution, they are expressed by the median (quartile spacing), that is, M (Q25 Q75), and the counting data are expressed by the number (percentage). The counting data were compared by Student’s t test or Wilcoxon's singed rank-sum tests (nonnormal distribution), and the classified variables were compared by chi-square tests or Fisher’s exact test. Pearson or Spearman correlation tests were used to evaluate the relationship between LAA-FV and various parameters. In addition, to evaluate the independent predictors of LAA-FV, multiple regression analysis was carried out. P < 0.05 was considered to be statistically significant. All statistical analyses were carried out using SPSS version 24.0 software (SPSS, Chicago, Illinois, USA).

### Ethics declarations

Patient studies were approved by the Ethics Committee of Affiliated Hospital of Xuzhou Medical University. All methods were implemented in accordance with relevant guidelines and regulations, and all enrolled patients signed informed consent forms.

## Results

### Characteristics of the study population

The characteristics of the patients are shown in Table 1. A total of 145 patients with symptomatic atrial fibrillation [76 with paroxysmal atrial fibrillation (52.41%) and 69 with nonparoxysmal atrial fibrillation (47.59%), including 6 with long-term persistent atrial fibrillation] were included, including 98 men (67.59%). The mean body mass index (BMI) was 25.24 ± 3.14 kg/m^2^, the left atrial volume index (LAVI) was 68.26 ± 23.97 ml/m^2^, the left atrial epicardial adipose tissue volume (LA-EAT) was 30.72 ± 11.81 ml, and the left atrial appendage flow rate (LAA-FV) was 47.59 ± 13.38 cm/s.

**Table 1:** Characteristics of study population.

	All patients
	n = 145
Age,years	60.81 ± 11.27
Male,n(%)	98 (67.59)
Body mass index, kg/m^2^	25.24 ± 3.14
Hypertension, n (%)	65 (44.83)
Diabetes mellitus, n (%)	23 (15.86)
Congestive heart failure, n (%)	19 (13.10)
Coronary disease, n (%)	28 (19.31)
Nonparoxysmal AF, n (%)	69 (47.59)
AF duration (month)	32.45 ± 56.73
LDL cholesterol, mg/dL	2.40 ± 0.84
eGFR (ml/min/m^2^)	100.40 ± 16.51
LV ejection fraction, %	59.20 ± 5.88
LAVI (mL/m^2^)	68.26 ± 23.97
LA-EAT volume (cm^3^)	30.72 ± 11.81
LAA morphology (C-W), n (%)	51 (35.17)
LAA flow velocity (cm/s)	47.59 ± 13.38

AF, atrial fibrillation; LDL-C, Low-density lipoprotein; LV, left ventricular; LAVI, left atrial volume index; LA, left atrial; EAT, Epicardial adipose tissue; LAA, Left atrial appendage; C-W, chicken-wing type.

### Comparison of baseline data and echocardiographic parameters

CARTO3 voltage mapping of the left atrium was performed in all cases under sinus rhythm, and an average of 864 marker points were obtained in each case. No complications occurred during the process of LA mapping. The voltage mapping results were used to divide the patients into a low voltage zone group (LVZ group) and a nonlow voltage group (non-LVZ group). Among them, 28 patients (19.31%) had left atrial LVZ. The clinical features, epicardial adipose tissue and echocardiographic parameters of the LVZ group and non-LVZ group were compared, and the results are shown in Table 2. Univariate analysis showed that the age of the LVZ group was older than that of the non-LVZ group. There were significant differences in terms of history of diabetes, left atrial volume index (LAVI), LA-EAT volume and left atrial appendage velocity (LAA-FV) between the two groups. In the LVZ group, there was more nonparoxysmal atrial fibrillation (P < 0.1), and the duration of atrial fibrillation was longer (P < 0.1). There was no significant difference in any other clinical or conventional echocardiographic parameters, including left ventricular ejection fraction, between the two groups (P > 0.05, Table 2).

**Table 2:** Comparison of baseline data and echocardiographic parameters.

	LVZ	No LVZ	P
	n = 28	n = 117	
Age, years	64.61 ± 9.23	59.89 ± 11.56	0.047
Male, n (%)	17 (60.71)	81 (69.23)	0.387
Body mass index, kg/m^2^	24.83 ± 3.22	25.26 ± 2.85	0.490
Hypertension, n (%)	12 (42.86)	52 (44.44)	0.879
Diabetes mellitus, n (%)	8 (28.57)	15 (12.82)	0.040
Congestive heart failure, n (%)	5 (17.86)	14 (11.97)	0.407
Coronary disease, n (%)	4 (14.29)	24 (20.51)	0.453
Nonparoxysmal AF, n (%)	18 (64.29)	51 (43.59)	0.049
AF duration (month)	50.80 ± 72.73	28.06 ± 51.06	0.056
LDL cholesterol, mg/dL	2.42 ± 0.87	2.33 ± 0.75	0.656
eGFR (ml/min/m^2^)	103.85 ± 13.95	99.58 ± 17.02	0.220
LV ejection fraction, %	59.04 ± 5.76	59.24 ± 5.93	0.870
LAVI (mL/m^2^)	83.42 ± 31.04	64.63 ± 20.50	< 0.001
LA-EAT volume (cm^3^)	37.35 ± 12.39	29.14 ± 11.14	0.001
LAA morphology (C-W), n (%)	9(32.14)	42(35.90)	0.371
LAA flow velocity (cm/s)	35.02 ± 10.78	50.60 ± 12.17	< 0.001

LVZ, Low Voltage Zone; AF, atrial fibrillation; LDL-C, Low-density lipoprotein; LV, left ventricular; LAVI, left atrial volume index; LA, left atrial; EAT, Epicardial adipose tissue; LAA, Left atrial appendage; C-W, chicken-wing type.

### Relationship between LAA-FV and relevant parameters

**Figure 5:**
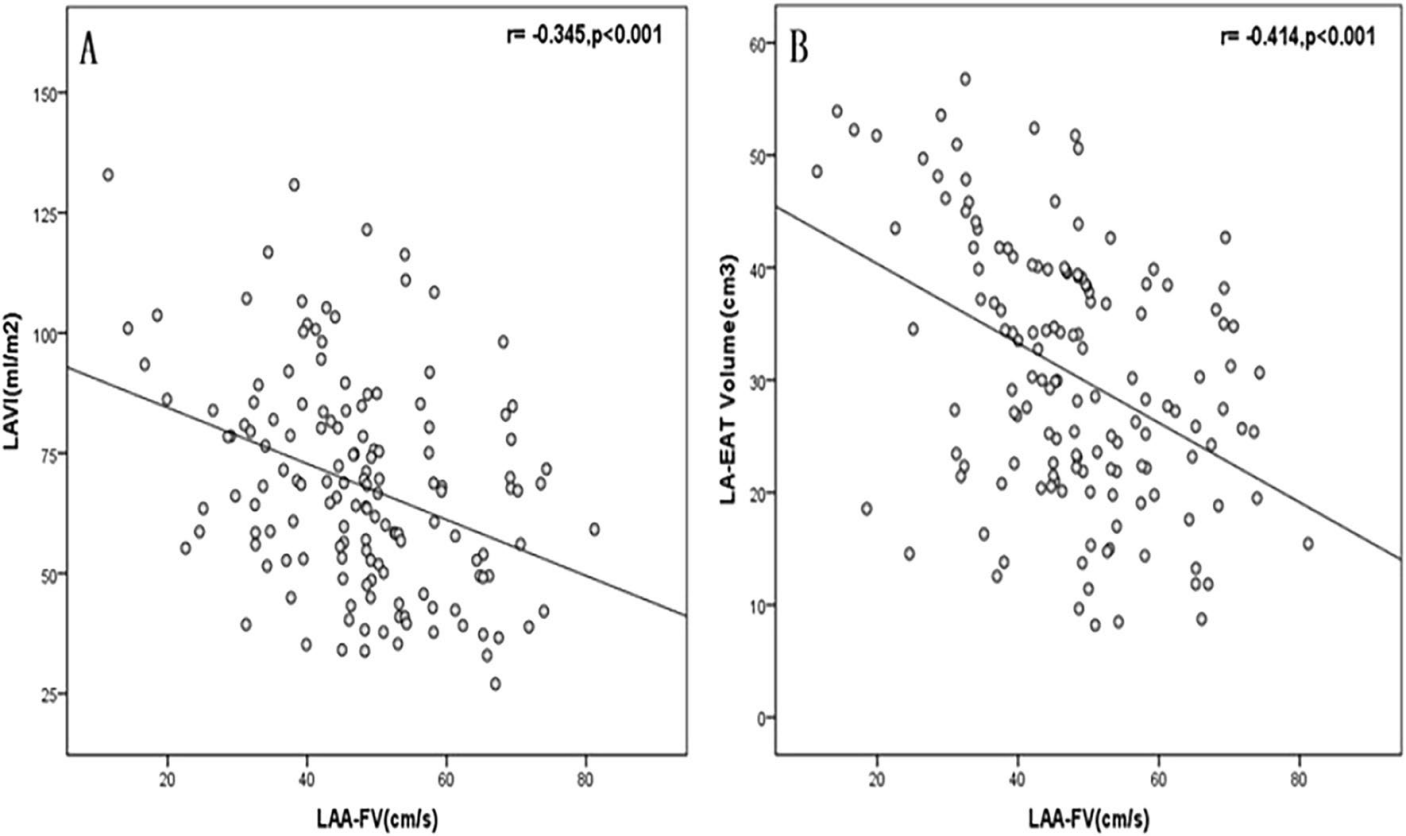
Correlation analysis showing that LAVI (**A**) and LA-EAT Volume (**B**) were significantly correlated with LAA-FV.

There were significant negative correlations between LAA-FV and left atrial volume index (LAVI) (r =  − 0.345, P < 0.001; Fig. 5A) and between LAA-FV and LA-EAT volume (r =  − 0.414, P < 0.001; Fig. 5B).

### Univariate and multiple linear regression analyses of LAA-FV

Univariate regression analysis showed that LAA-FV was lower in the patients with an LVZ (with low voltage and without low voltage; 35.02 ± 10.78 cm/s vs. 50.60 ± 12.17 cm/s, P < 0.001) and nonchicken wing type (nonchicken wing type and chicken wing type; 44.82 ± 11.91 cm/s vs. 52.71 ± 14.53 cm/s, P = 0.001). The LAA-FV of the persistent AF group was lower than that of the paroxysmal AF group (42.15 ± 12.53 cm/s vs. 52.54 ± 12.14 cm/s, P < 0.001). In addition, a lower LAA-FV was significantly correlated with the left atrial volume index (LAVI) and the LA-EAT volume (p ≤ 0.001). In a multiple linear regression model, the left atrial LVZ (β = − 0.311, P < 0.001), LA-EAT volume (β = − 0.256, P < 0.001), left atrial appendage shape (β = − 0.216, P < 0.001), LAVI (β = − 0.153, 0.041), and type of atrial fibrillation (paroxysmal vs. persistence) (β = − 0.146, 0.048) were independent predictors of LAA-FV (Table 3).

**Table 3:** Univariate linear regression analyses and multivariate linear regression analysis of Left Atrial Appendage Flow Velocity.

Variables	Univariate		Multivariate	
	*B*	*p* value	*B*	*p* value
Age,years	− 0.097	0.246		
Male,n(%)	0.044	0.596		
Body mass index, kg/m^2^	− 0.159	0.131		
Hypertension, n (%)	− 0.128	0.126		
Diabetes mellitus, n (%)	− 0.044	0.601		
Congestive heart failure, n (%)	− 0.134	0.107		
Coronary disease, n (%)	0.005	0.952		
Nonparoxysmal AF, n (%)	− 0.389	< 0.001	− 0.146	0.048
AF duration (month)	− 0.074	0.378		
LDL cholesterol, mg/dL	− 0.001	0.992		
eGFR (ml/min/m^2^)	0.010	0.901		
LV ejection fraction, %	0.162	0.051	0.044	0.507
LAVI (mL/m^2^)	− 0.345	< 0.001	− 0.153	0.041
LA-EAT volume (cm^3^)	− 0.414	< 0.001	− 0.256	< 0.001
LAA morphology (C-W), n (%)	− 0.282	0.001	− 0.216	0.001
LAZ, n (%)	− 0.461	< 0.001	− 0.311	< 0.001

AF, atrial fibrillation; LDL-C, Low-density lipoprotein; LV, left ventricular; LAVI, left atrial volume index; LA, left atrial; EAT, Epicardial adipose tissue; LAA, Left atrial appendage; C-W, chicken-wing type; LVZ, low-voltage zone.

## Discussion

In recent years, increasing attention has been given to the relationship between epicardial adipose tissue and atrial fibrillation (AF). Venteclef et al.^20^ reported that human adventitia adipose tissue (EAT) caused fibrosis of atrial cardiomyocytes in rats and promoted fibroblasts to differentiate into myofibroblasts. At present, the clinical detection methods for myocardial fibrosis are limited. Myocardial fibrosis is mainly detected through endocardial biopsy, late gadolinium enhancement cardiac magnetic resonance (LGE-CMR) and intracardiac voltage mapping. In this study, left atrial voltage mapping was performed during radiofrequency ablation, and patients with atrial fibrillation were divided into an LVZ group and a non-LVZ group. Compared with the non-LVZ group, the LAA-FV in the LVZ group was lower. This is consistent with the results of previous studies that show that there may be a correlation between atrial fibrosis and left atrial appendage velocity^13,14^. At present, TEE is the most widely used method to measure the function of the LAA, and it is considered the gold standard, but it is semi-invasive. We hope to find a non-invasive and reliable index to evaluate left atrial appendage function. In this study, it was found that left atrial LVZ, LA-EAT volume, left atrial appendage shape, left atrial volume index and type of atrial fibrillation (paroxysmal vs. persistence) were independent predictors of LAA-FV.

Yuichi Hori et al.^21^ found that patients with low LAA-FV (20 cm/s) were characterized by the presence of left anterior wall LVZ and high CHA_2_DS_2_VASc scores. SHIH-HSIENSUNG et al.^22^ found that the larger the left atrium was, the larger the LVZ of the left atrium, and there was a significant correlation between the LVZ of the left atrium and the emptying velocity of the LAA. These results are consistent with our research. A possible reason is that atrial structural remodeling characterized by atrial fibrosis is the core of the maintenance mechanism of atrial fibrillation^23^. Left atrial electrical remodeling and fibrosis cause greater left atrial pressure, which is more likely to increase the afterload of the left atrial appendage and cause systolic dysfunction, therefore resulting in a decrease in LAA-FV. Another possible reason is that this structural remodeling also occurs in the left atrial appendage^24^. LAA-FV can reflect changes in left atrial function, including contraction, stunning, fibrosis, etc., so the LAA-FV may represent the severity of the left atrial remodeling^25^.

In a previous study, the types of atrial fibrillation were divided into paroxysmal atrial fibrillation, persistent atrial fibrillation and long-term persistent atrial fibrillation, and the results showed that LAA-FV decreased gradually^26^. The results of this study are consistent with the above studies, and the mechanism may be as follows: first, during TEE, the heart rhythm of patients with paroxysmal atrial fibrillation shows a sinus rhythm, while that of patients with persistent atrial fibrillation shows an atrial fibrillation rhythm. Thus, the emptying time of the left atrium and left atrial appendage is shortened, and rapid irregular electrical activity can significantly affect the active contractile force of the left atrial appendage muscle and decrease the velocity of the left atrial appendage.

Previous studies have shown that LAAFV and LAA morphology are closely related. Lee JM et al.^27,28^found that the LAA orifice enlargement or a chicken wing type was closly related with the decreased flow velocity of LAA. The results of this study are consistent with the above studies. This may be because the chicken wing-type LAA has a higher muscle mass to contract the left atrial appendage^29^. Previous studies have shown that there is a significant negative correlation between the left atrial diameter and LAA-FV^30,31^. LAVI was used in this study because the use of the LA volume can more accurately identify structural atrial remodeling that involves the left atrial low-voltage matrix than the LA diameter. The results showed that LAVI was negatively correlated with LAA-FV and was an independent predictor of LAA-FV. The left atrial appendage is thought to act as a decompression chamber during left ventricular contraction and other increases in left atrial pressure^32^. LAA-FV may represent the full functionality of LA and indicate the severity of LA refactoring^33^. Therefore, dilatation of the left atrium and an increase in LAVI leads to remodeling of the left atrial appendage and a decrease in LAA-FV. This may also explain why some scholars believe that the risk of stroke increases with increasing left atrial size^34^.

A previous study^35^ reported that the capacity of EAT may be related to the function of the left atrial appendage. However, that study did not investigate echocardiographic parameters. Yamaguchi et al.^36^ evaluated the thickness of EAT by TTE. The results showed that the thickness of the EAT was negatively correlated with the filling and emptying velocity of the left atrial appendage. Because the thickness of the EAT varies greatly in different parts and the measurement repeatability is poor, this study improves the accuracy by measuring the EAT volume. It was found that the adipose tissue volume of the left atrium was an independent predictor of LAA-FV. The explanation may be as follows: First, EAT can cause atrial myocardial fibrosis through various pathways. Haemers et al.^37^proposed that EAT could lead to progressive fibrosis of the adjacent atrial myocardium through fatty infiltration. Venteclef et al.^20^ found that EAT is the main source of activin A, and activin A may be the main factor mediating the pro-fibrotic effect of EAT. A recent study by Patel et al.^38^ found that EAT can induce myocardial fibrosis by activating the renin–angiotensin–aldosterone system (RAAS), especially angiotensin II (Ang II). Previous studies^39–41^have confirmed that EAT can secrete inflammatory factors including interleukin-1 (IL-1), interleukin-6 (IL-6), C-reactive protein (CRP), tumor necrosis factor-α (TNF-α), monocyte chemoattractant protein-1 (MCP-1), nerve growth factor (NGF) and other inflammatory factors through endocrine and paracrine pathways. Under the action of these factors, myocardial inflammatory response is caused, which leads to myocardial fibrosis. As mentioned above, atrial fibrosis can lead to decreased LAAFV. Second, EAT is also closely related to oxidative stress. Salgado-Somoza et al.^42^ obtained EAT from 55 patients undergoing cardiac surgery and detected oxidative stress indicators such as reactive oxygen species (ROS) in EAT tissue. The results suggest that EAT has higher levels of oxidative stress in patients with cardiovascular disease compared to other adipose tissues. Dudley et al.^43^ established an atrial fibrillation model by rapid atrial pacing in pigs. The results showed that the level of oxidative stress in LAA tissues was significantly increased. Therefore, we speculate that the effect of EAT on LAA function may be related to oxidative stress. Oxidative stress can lead to cellular damage, impair myofibril energetics, and lead to myocardial contractile dysfunction, resulting in a decrease in LAAFV^44^.

## Limitations

There are some limitations in this study. First, this study is a retrospective, single-center, small sample study, so it is necessary to conduct a prospective study on a larger cohort. Second, when measuring LA-EAT, semiautomatic methods are used to manually outline the epicardium, which may lead to differences in LA-EAT evaluation. In this study, two experienced imaging physicians separately performed the LA-AT measurement to reduce this measurement artifact. Third, the specific mechanisms of LA-EAT and LAA-FV are still unclear, such as EAT-induced fibrosis, inflammation, and fat infiltration, which need to be studied further.

## Conclusion

In NV-AF patients, the increase in LA-EAT is related to the decrease in LAA-FV. Currently, there are some limitations to CHA2DS2-VASc score to assess stroke risk in patients with NV-AF, which may be related to the fact that the score does not reflect the effect of cardiac structure and function on thrombosis. EAT may be able to compensate for the shortcomings of the CHA2DS2-VASc score.

## Data Availability

The raw data supporting the conclusions of this manuscript will be made available by the authors, without undue reservation, to any qualified researcher.
